# The Adult Patient with Eisenmenger Syndrome: A Medical Update after Dana Point  Part II: Medical Treatment - Study Results

**DOI:** 10.2174/157340310793566163

**Published:** 2010-11

**Authors:** Siegrun Mebus, Ingram Schulze-Neick, Erwin Oechslin, Koichiro Niwa, Pedro T Trindade, Alfred Hager, John Hess, Harald Kaemmerer

**Affiliations:** 1 Department of Pediatric Cardiology and Congenital Heart Disease, Deutsches Herzzentrum München, Technische Universität München, München, Germany; 2 UK Service for Pulmonary Hypertension in Children, Cardiac Unit, Great Ormond Street Hospital, London, WC1N 3JH, United Kingdom; 3 Congenital Cardiac Centre for Adults, University Health Network/Toronto General Hospital/Peter Munk Cardiac Centre, 585 University Avenue, Toronto, ON. M5G 2N2, Canada; 4 Department of Pediatrics, Chiba Cardiovascular Center, 575 Tsurumai, Ichihara, Chiba 290-0512, Japan; 5 Department of Cardiology, University Hospital Zurich, Rämistrasse 100, 8091 Zürich, Switzerland; 6 Department of Cardiology, University of Vienna, Währinger Gürtel 18-20, 1090 Wien, Austria

**Keywords:** Cardiovascular diseases, adult congenital heart defects, pulmonary hypertension, Eisenmenger syndrome, follow-up studies, Competence Network for Congenital Heart Defects.

## Abstract

Eisenmenger syndrome is the most severe form of pulmonary arterial hypertension and arises on the basis of congenital heart disease with a systemic-to-pulmonary shunt. Due to the chronic slow progressive hypoxemia with central cyanosis, adult patients with the Eisenmenger syndrome suffer from a complex and multisystemic disorder including coagulation disorders (bleeding complications and paradoxical embolisms), renal dysfunction, hypertrophic osteoarthropathy, heart failure, reduced quality of life and premature death.

For a long time, therapy has been limited to symptomatic options or lung or combined heart-lung transplantation. As new selective pulmonary vasodilators have become available and proven to be beneficial in various forms of pulmonary arterial hypertension, this targeted medical treatment has been expected to show promising effects with a delay of deterioration also in Eisenmenger patients. Unfortunately, data in Eisenmenger patients suffer from small patient numbers and a lack of randomized controlled studies.

To optimize the quality of life and the outcome, referral of Eisenmenger patients to spezialized centers is required. In such centers, specific interdisciplinary management strategies of physicians specialized on congenital heart diseases and PAH should be warranted.

This medical update emphasizes the current diagnostic and therapeutic options for Eisenmenger patients with particularly focussing on the medical treatment and corresponding study results.

## 1. MEDICAL MANAGEMENT - STUDY RESULTS

Timing of treatment in Eisenmenger patients is a sensitive issue, particularly in stable patients. There exist no studies or guidelines on this subject. Especially for the stable patient, the “noli-me-tangere” ruling the treatment strategies for Eisenmenger patients still holds -at least to some extend-with respect to the delicate balance of the many variables, in spite of the available specific pulmonary vasodilators. On the other hand, treatment may indeed appear indicated because of reduced exercise tolerance, increasing cyanosis, or increasing signs of heart failure [1]. 

However, it has to be kept in mind, that reduction of right ventricular afterload by pulmonary vasodilators is impossible in univentricular function. In other subtypes of congenital heart defects with Eisenmenger reaction (ER), the effect of these drugs may be more pronounced on the systemic vasculature, leading to systemic vasodilatation and, consecutively, in increased cyanosis. If indeed the pulmonary vascular system preferentially vasodilates, the increased pulmonary blood flow may cause left heart failure and pulmonary edema.

### 1.1. Anticoagulation

Systemic anticoagulation in patients with Eisenmenger syndrome (ES), remains controversial as randomized controlled data are lacking [2]. Eisenmenger patients suffer from multivarious haemostatic abnormalities. On the one hand, ES is associated with a variety of procoagulant biochemical aberrations [3]. Thromboembolic events in the pulmonary circulation occur in approximately 20% of these patients [4]. Therefore, anticoagulation seems logical for the prevention and treatment of thrombosis [5].

Otherwise, Eisenmenger patients are at increased risk of fatal and life threatening bleeding complications, particularly significant hemoptysis [4]. Besides a deficiency of coagulation factors and abnormal fibrinolysis, there is thrombocytopenia with platelet malfunction. 

Moreover, it is difficult to assess the optimal level of anticoagulation by routine laboratory tests (see below). So far, systemic anticoagulation and the use of platelet aggregation inhibitors is still not generally recommended in Eisenmenger patients.

### 1.2. Long-Term Oxygen-Therapy

The use of long-term oxygen supplementation in adult patients with ES is controversial. There are few data and only one published study with a prospective controlled design [6]. Although some patients (e.g. with intense hypoxemia, dyspnea at rest and loss of vital capacity) might subjectively benefit from oxygen supplementation, the risk and side-effects of this therapy (e.g. desiccation of nasal mucosa, epistaxis, sleep disturbance, etc.) should be taken into account. In addition, the above-mentioned trial [6] showed no impact of nocturnal oxygen therapy on exercise capacity, natural history and survival of the patients within a follow up period of 2 years. 

According to the guidelines, supplemental oxygen is a general recommendation for PAH patients. The routine use of supplemental oxygen at home is not recommended for Eisenmenger patients, the use should be at the treating physician’s discretion.

### 1.3. Nitric Oxide (NO)

Nitric oxide (NO) is a potent and selective pulmonary arteriolar vasodilator produced in endothelial cells [7]. It has a crucial function in regulating basal vascular resistance and may also affect platelets and vascular endothelial remodeling. Studies have shown inhaled NO to reduce pulmonary vascular resistance with minimal systemic effects in patients with ES and acute pulmonary hypertensive states of other etiologies [8, 9]. Amongst these, beneficial hemodynamic effects have been anecdotally described in children with ES [10].

Due to the need of continuous inhalation, NO does not play any role in the long-term therapy and therefore only rare patients have been treated chronically. However, NO has an important role in the acute post-operative therapy. In addition, NO is established for the assessment of pulmonary vascular reactivity to identify patients with advanced pulmonary hypertension and ES, who could benefit from sustained vasodilator treatment [11].

### 1.4. Calcium channel blockers (CCBs)

The use of high doses of oral calcium channel blockers in pulmonary arterial hypertension remains very limited. There is a lack of randomized controlled trials and the available data are restricted to patients with iPAH. 

Even though favorable clinical and prognostic effects of high doses of CCB drugs have been suggested [12], these effects were only demonstrable in patients with iPAH with an acute response to vasodilator testing (“responders”) [13, 14]. Hence, merely a minority subgroup of patients appears to benefit from this therapy. By contrast, CCB use in associated forms of PAH has been discouraged, particularly in patients who do not fulfill the criteria of hemodynamic responders, e.g. Eisenmenger patients [15].

As the effects of CCB are not restricted to the pulmonary circulation, vasodilator therapy with CCBs could even cause complications. In Eisenmenger patients, systemic vascular resistance could be lowered more than pulmonary vascular resistance, thus increasing the right-to-left shunt with worsening cyanosis and hypotension. For this reason, empiric CCB therapy in adult Eisenmenger patients is not recommended [15]. 

### 1.5. Endothelin-1 (ET-1) Receptor Antagonists

ET-1 is a powerful vasoconstrictor with elevated concentrations in the plasma and lung tissue of patients with PAH. It plays a key role in the pathogenesis of PAH including *in vitro* effects on proliferation, fibrosis and inflammation. As increased ET-1 plasma levels have been correlated with the severity and prognosis of PAH [16], the ET-1 pathway represents an important treatment target.

#### Bosentan

Bosentan is a non-selective endothelin receptor antagonist with dual activity on both ET_A_ and ET_B_ receptors and thus completely blocking the activity of ET-1. It is the first oral drug of this medical category, which has been approved by the FDA and EMEA in 2002 as orphan-drug for the treatment of pulmonary hypertension, and currently also for mildly symptomatic patients [17]. Furthermore, since July 2009 Bosentan is the only approved drug for the treatment of PAH in children, as there is a paediatric formulation approved for children with an age of at least 2 years [18, 19].

Particularly for the treatment of Eisenmenger patients, several case series and uncontrolled studies have been published, consistently demonstrating an improvement in exercise capacity and hemodynamics with bosentan treatment [20-23]. 

BREATHE-5 was designed as the first randomized, placebo-controlled and double-blind trial exclusively enrolling Eisenmenger patients. After a treatment period of 16 weeks receiving bosentan, patients showed a significant improvement in hemodynamics and 6 minute walking distance (6 MWD), without adversely affecting systemic arterial oxygen saturation [24]. In the BREATHE-5 open-label extension study, improvement in exercise capacity was maintained up to 40 weeks [25]. So far, the results of this follow-up were confirmed in two prospective, uncontrolled and open-label studies, which demonstrated an initial persistent improvement of objective exercise capacity, but a decline after one year [26] with reduction to baseline levels after two years [27]. In children, deterioration seemed to be more progressive, whereas in adult patients with the ES, the improvement appeared to last longer. However, these data have to be evaluated carefully due to the limited long-term experience, small subject groups and uncontrolled trial designs. In addition, natural progression of the disease cannot be distinguished from a possible tachyphylaxis. 

Overall, bosentan related side effects include dose-dependent elevation of hepatic transaminases, edema and systemic hypotension. Bosentan may also interfere with the action of hormonal contraceptives.

In summary, based on the BREATHE-5 study as well as clinical evidence, bosentan seems to be safe and effective in PAH related to CHD, showing improvement in hemodynamic parameters, exercise capacity and functional class. Further experiences with bosentan in another large cohort of Eisenmenger patients, conducted by the German Competence Network for Congenital Heart Defects, are expected in the near future. Bosentan is currently approved for the treatment of severe PAH related to the ES. 

#### Sitaxsentan

Sitaxsentan is a potent and highly selective ET_A_ receptor antagonist with a distinctive oral bioavailability and a half-life of up to 7 hours, allowing effective once daily oral dosing. Since October 2006, sitaxsentan is the first ET_A_ receptor antagonist approved for the treatment of PAH. There are few randomized-controlled studies (Table **1**), demonstrating improvements in exercise capacity, hemodynamic parameters, WHO functional class and clinical events in patients with PAH of different etiologies [28, 29]. Similar to other pharmaceutical agents, the above-mentioned trials of sitaxsentan were predominantly focused on iPAH, while only a minority of patients suffered from PAH associated with CHD. Available data show that sitaxsentan has a lower incidence of hepatic toxicity than bosentan, but affects the metabolism of Warfarin.

First long-term-results of a one year prospective, observational and open-label study have recently been published, suggesting sitaxsentan therapy to be safe and efficacious for patients with PAH. In an uncontrolled study arm, there was a suggestion that patients treated with sitaxsentan demonstrated a longer time to clinical worsening than those in the bosentan group [30]. Unfortunately, there was no subanalysis of the data for the various subgroups of PAH, such as PAH related to CHD. Therefore no conclusion can be drawn concerning Eisenmenger patients.

The experience with sitaxsentan specifically in Eisenmenger patients remains limited. Currently, there are few anecdotal reports and no randomized trials. Only Rosen-zweig [31] reported on a retrospective data analysis of sitaxsentan treatment in 14 Eisenmenger patients. After a follow-up period of up to 13 months, treatment appeared to be safe without a significant decrease in oxygen saturation. There was an improvement in hemodynamics and pulmonary to systemic vascular resistance ratio suggesting pulmonary selectivity. 6 MWD improved, but this was not statistically significant. Limited data underscore the need for further studies in patients with the ES.

#### Ambrisentan

Ambrisentan, another selective ET_A_ receptor antagonist, has recently been approved for the treatment of PAH in WHO class II and III. ARIES-1 and ARIES-2 [32] (Table **1**) demonstrated beneficial effects on exercise capacity (6 MWD), WHO class and time to clinical worsening. Regrettably, no patients with PAH related to CHD were included in the trials and currently there are no further data available for Ambrisentan treatment in such patients. Therefore, no conclusion can be drawn concerning the effect of Ambrisentan in patients with ES.

### 1.6. Phosphodiesterase-5 (PDE-5) inhibitors

Over the last years, PDE-5 inhibitors have been approved for the treatment of erectile dysfunction. As Type 5 PDE receptors are located predominantly in the penile and pulmonary vasculature, PDE-5 inhibitors are a potential group of medications for the treatment of pulmonary arterial hypertension. Currently, there are three different PDE-5 inhibitors, which have been studied in patients with PAH (Table **2**).

Sildenafil and tadalafil^1^ have shown beneficial effects on pulmonary selectivity and arterial oxygenation.

SUPER-1, the pivotal study of **Sildenafil** [35], was the first large prospective multicenter blinded and controlled study demonstrating an improvement in exercise capacity, as assessed according to the six-minute walking test, functional class and hemodynamics in PAH patients. Therefore a treatment dose of 20 mg three times per day (TID) has been approved by the FDA and EMEA. Unfortunately, there is no sufficient experience for long-term-treatment. In those patients treated long-term, higher doses were used. 

Similar to other vasodilators, sildenafil showed promising effects in patients with iPAH. Recently, favorable effects have been reported in patients with the ES. However, so far there are only a number of individual cases, several case series [36], observational studies and few randomized placebo-controlled trials [37] with increasing evidence for sildenafil in Eisenmenger patients. 

In summary, preliminary results have demonstrated that sildenafil is safe and improved symptomatic status, functional class, exercise capacity (6 MWD and exercise duration) and pulmonary hemodynamic parameters in patients with severe pulmonary hypertension related to the ES [38-40]. As these data are based on relatively small subject groups, appropriate studies with larger cohorts are necessary, and are currently being conducted by the German Competence Network for Congenital Heart Defects.

**	Tadalafil** is another PDE-5 inhibitor reported to affect PAH in patients with the ES in an observational study. In one study from India, oxygen saturation and the mean functional class improved after a 12-weeks treatment with tadalafil in selected symptomatic Eisenmenger patients [41]. At this time, the limited available data show tadalafil to be safe and effective in these patients, even though further investigations are required. 

### 1.7. Prostacyclin and Prostacyclin Analogs

Prostanoids can be administered by continuously intravenous or subcutaneous infusion, by inhalation and orally. Due to their active profile with vasodilatory, antiproliferative, anti-inflammatory and anticoagulant effects, they are suitable drugs for the treatment of PAH (Table **3**). In current treatment algorithms for patients with WHO Functional Class III and IV, prostacyclin analogs are indicated particularly in patients with right heart failure. 

#### Epoprostenol

Prostacyclin is a potent endogenous vasodilator produced in the vascular endothelium. Epoprostenol was the first synthetic prostacyclin analog, which became standard therapy of severe PAH in many countries. Due to a short half-life of a few minutes, continuous i.v.-infusion of epoprostenol is required, which exposes patients to significant side-effects and associated risks. Epoprostenol is well studied in patients with iPAH, and randomized controlled trials have shown improvements in exercise capacity, quality of life and hemodynamics [33, 42]. In patients with PAH caused by congenital cardiac lesions in whom conventional therapy has failed, long-term prostacyclin therapy has shown amelioration in hemodynamics and quality of life following one year of treatment [43, 44], even though there were serious adverse events reported, including cerebrovascular accidents. Another case series studied i.v.-epoprostenol in Eisenmenger patients, showing improved oxygenation and 6 MWD [45]. Unfortunately, the data are limited and there is a lack of randomized controlled trials in patients with ES. Although Eisenmenger patients might also benefit from this treatment option with intravenous epoprostenol, there exist limited data on the efficacy and safety [43].

#### Iloprost

Intravenous iloprost is a very stable prostacyclin analog and therefore an alternative to i.v.-prostacyclin. Inhaled iloprost has been studied extensively and it has been assumed that it is pulmonary selective, thus minimizing systemic side effects [46]. With a serum half-life of up to 25 minutes after inhalations, inhaled iloprost has to be administered 6-8 times per day. One randomized controlled trial with a 12-weeks treatment period with inhaled iloprost showed beneficial effects in terms of hemodynamics, exercise capacity, symptoms and clinical events [47] in patients with iPAH. So far, the efficacy in adult Eisenmenger patients has not yet been studied.

#### Treprostinil

Treprostinil is a stable prostacyclin analog with a half-life of three hours, currently available for subcutaneous and intravenous application. The effects of its continuous subcutaneous administration were studied in a large randomized controlled trial with patients suffering from iPAH (58%), PAH related to connective tissue disease (19%) and PAH caused by CHD (24%). There were beneficial effects on exercise capacity, hemodynamics and clinical events [48], but notably a high frequency of site pain limiting subcutaneous administration. For intravenous application, only few data exist with minuscule evidence for PAH in relation to congenital cardiac lesions [49]. Long-term use of treprostinil has resulted in similar survival benefits as i.v. epoprostenol [50]. 

#### Beraprost

Beraprost is the first orally active prostacyclin analog that is approved for treatment of iPAH only in Japan. After oral administration, peak concentrations are reached after 30 minutes. 

There are two randomized controlled trials with beraprost and a relatively large trial size of 130 and 116 patients, suffering from iPAH and PAH associated with connective tissue disease and CHD, respectively. In the first trial, patients were randomized to receive the maximal tolerated dose of beraprost or placebo for 12 weeks [51]. Subgroup data analysis demonstrated that beraprost improved exercise capacity particularly in patients with iPAH, while those with associated conditions showed no significant changes. Moreover, there were no relevant beneficial effects in cardiopulmonary hemodynamics and WHO functional class. The second trial studied the long-term effects of beraprost treatment up to one year [52]. During earlier phases of treatment, data suggest less disease progression persisting up to 6 months, but after one year, there was no longer any difference between the beraprost and the placebo groups. Therefore, beraprost does not play a crucial role in the treatment of PAH related to congenital cardiac lesions and ES. 

### 1.8. Combination Therapy

Combination of various drugs may have synergistic effects through interaction of different pathobiological pathways [53-56]. Data are limited, but combination therapy may be considered for symptomatic patients who failed to improve with first-line, monodrug treatment. Several small trials with limited recruiting numbers are under way, but so far there are only anecdotal reports and different single-center experiences of Eisenmenger patients treated with a combination of specific anti-pulmonary hypertensive agents [57, 58]. It could be expected that a combination therapy as goal oriented treatment in Eisenmenger patients may be a more beneficial standard therapy in the future, but caution should be advised to unknown interactions of the agents with regard to potential toxicity. Currently, controlled data justifying the use of combination therapy in ES patients are lacking.

### 1.9. Outlook for the Future

Future studies need to define valid and practical endpoints that are customized to ES patients. While what we learn and achieve here may serve as a model for pulmonary vascular disease in CHD in general. It will be an important question, whether drugs affecting remodeling processes will be usable. 

## Figures and Tables

**Table 1.** Controlled Clinical Trials with Entothelin-1 Receptor Antagonists in Patients with PAH. (Table Adapted from Galie [33])

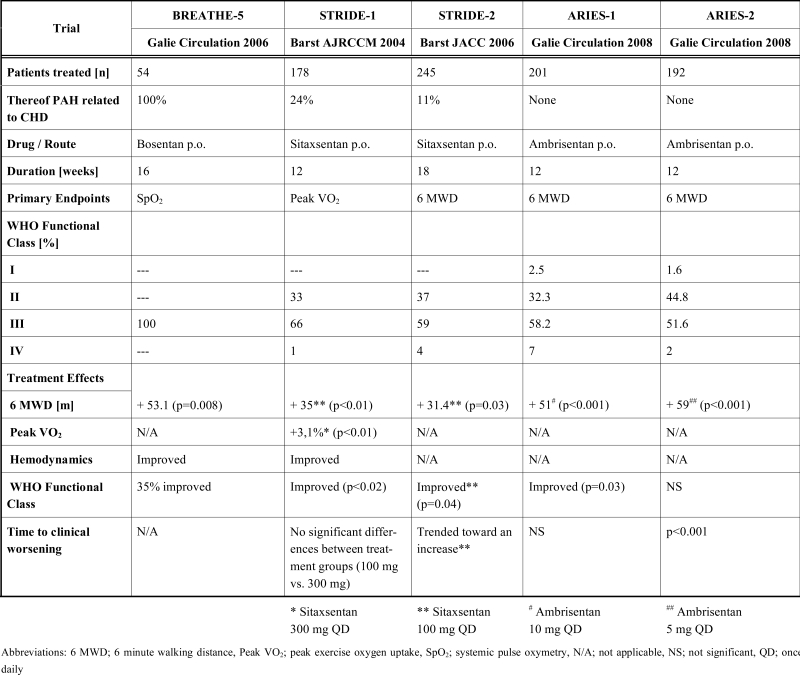

**Table 2.** PDE-5 Inhibitors in Patients with PAH [34]

	Sildenafil	Vardenafil	Tadalafil
**T _max_ [min]**	60	40 - 45	75- 90
**T _½_ [h]**	~ 3,5	~ 3,5	17,5
**PVR/SVR**	Decrease	---	Decrease
**paO_2_**	significant improvement	---	---

Abbreviations: T max; time to peak hemodynamic effects, T ½; mean half-life, PVR/SVR; pulmonary to systemic vascular resistance ratio, paO_2_; arterial oxygenation

**Table 3.** Controlled Clinical Trials with Prostacyclin Analogs in Patients with PAH. (Table Adapted from Galie [33])

Trial	Treprostinil	ALPHABET	Beraprost-LT	AIR
	Simonneau AJRCCM 2002	Galie JACC 2002	Barst JACC 2003	Olschewski N Engl J Med 2002
**Patients [n]**	469	130	116	203
**Thereof PAH related to CHD**	23%	18%	16%	None
**Drug / Route**	Treprostinil s.c.	Beraprost p.o.	Beraprost p.o.	Iloprost inh.
**Duration [months]**	3	3	12	3
**Primary Endpoints**	6 MWD	6 MWD	Disease progression	6 MWD
			Peak VO_2_	WHO Functional Class
**WHO Functional Class [%]**				
**I**	---	---	---	---
**II**	11	49	53	59
**III**	82	51	47	41
**IV**	7	---	---	---
**Treatment Effects**				
**6 MWD [m]**	+ 16 (p=0.006)	+ 25 (p=0.036)	+ 23 (p=0.18)	+ 36 (p=0.06)
**Peak VO_2_**	N/A	N/A	Trend to increase (NS)	N/A
**Hemodynamics**	Improved	No Change	No Change	Improved
**WHO Functional Class**	N/A	NS	58% unchanged (p=0.155)	65% unchanged
**Time to clinical worsening**	N/A	N/A	N/A	N/A

Abbreviations: 6 MWD; 6 minute walking distance, Peak VO_2_; peak exercise oxygen uptake, N/A; not applicable, NS; not significant

## Footnotes

1 Galie N, Brundage H, Ghofrani A, Oudiz RJ, Simonneau G, Beardsworth A, Chan M, Barst RJ. Tadalafil therapy in pulmonary arterial hypertension: results of a randomized, double-blind, placebo-controlled, phase III study. European Heart Journal 2008; 29 (Abstract Supplement): 519
